# Crohn’s Disease in Clinical Remission Is Marked by Systemic Oxidative Stress

**DOI:** 10.3389/fphys.2019.00499

**Published:** 2019-04-26

**Authors:** Arno R. Bourgonje, Julius Z. H. von Martels, Marian L. C. Bulthuis, Marco van Londen, Klaas Nico Faber, Gerard Dijkstra, Harry van Goor

**Affiliations:** ^1^Department of Gastroenterology and Hepatology, University Medical Center Groningen, University of Groningen, Groningen, Netherlands; ^2^Department of Pathology and Medical Biology, University Medical Center Groningen, University of Groningen, Groningen, Netherlands; ^3^Department of Internal Medicine, Division of Nephrology, University Medical Center Groningen, University of Groningen, Groningen, Netherlands

**Keywords:** Crohn’s disease, free thiols, oxidative stress, redox status, disease activity, biomarker

## Abstract

**Introduction:** Crohn’s disease (CD) is characterized by chronic and relapsing inflammation of the gastro-intestinal tract. It is assumed that oxidative stress contributes to CD pathogenesis, but systemic biomarkers for oxidative stress in CD are not yet identified. A reduction in free thiol groups in plasma proteins (“plasma free thiols”) reflects systemic oxidative stress since they are prime substrates for reactive oxygen species. Here, we determined the concentrations of plasma free thiols in CD patients and healthy controls and studied the putative correlation with disease parameters.

**Methods:** Free thiols were quantified in plasma of patients with CD in clinical remission [according to the Harvey Bradshaw Index (HBI)] and healthy controls and adjusted for plasma albumin. Albumin-adjusted free thiol concentrations were analyzed for associations with clinical and biochemical disease markers.

**Results:** Mean plasma free thiol concentrations were significantly lower in patients with CD (*n* = 51) compared to healthy controls (*n* = 27) (14.7 ± 2.4 vs. 17.9 ± 1.8 μmol/g albumin; *P* < 0.001). Patients with CD with above-average free thiols had significantly lower CRP levels (median 1.4 [interquartile range] [0.4; 2.6] vs. 3.6 [0.6; 7.0] mg/L; *P* < 0.05) and BMI (23.6 ± 4.8 vs. 27.1 ± 5.2 kg/m^2^; *P* < 0.05). Patients with CD having solely colonic disease demonstrated markedly reduced plasma free thiol concentrations compared to patients with ileocolonic involvement (13.2 ± 1.8 vs. 15.2 ± 2.2 μmol/g; *P* < 0.05). Finally, plasma free thiol concentrations negatively correlated with biomarkers of inflammation, including hsCRP, SAA, IL-17A (all *P* < 0.05), and VEGF.

**Conclusion:** Plasma free thiols are reduced in patients with CD in clinical remission compared to healthy controls. Thus, subclinical CD disease activity is reflected by systemic oxidative stress and plasma free thiols may be a relevant therapeutic target and biomarker to monitor disease activity in CD.

## Introduction

Crohn’s disease (CD) is an inflammatory bowel disease (IBD) characterized by chronic transmural intestinal inflammation that can affect any part of the gastro-intestinal tract (Sartor, 2008). Most patients follow a disease course of alternating exacerbations and remissions, which are difficult to predict and adequately treat (Cosnes et al., 2011). CD has a complex, multifactorial origin, eventually leading to an inappropriate and uncontrolled immune response with impaired gut mucosal homeostasis (Fiocchi, 1998; Abraham and Cho, 2009). At the mucosal level, IBD is characterized by chronic infiltration of various activated inflammatory cells, including polymorphonuclear cells, eosinophils and plasma cells (Kruidenier and Verspaget, 2002). Besides, even in clinical remission, subclinical intestinal inflammation is present in a large proportion of CD patients (Cosnes et al., 2011).

Oxidative stress has been implicated to play a pivotal role in CD pathogenesis, and may be a key effector mechanism leading to cellular/molecular damage and tissue injury (Pereira et al., 2015; Guan and Lan, 2018). Oxidative stress is characterized by increased production of reactive oxygen species (ROS). Accumulating evidence indicates that chronic intestinal inflammation in IBD is intimately associated with increased systemic levels of oxidative stress and enhanced ROS production, however, this evidence is predominantly derived from tissue analysis (Keshavarzian et al., 1992; Tanida et al., 2011; Zhu and Li, 2012; Pereira et al., 2015). Furthermore, plasma and mucosal antioxidant defense components that are necessary to neutralize ROS are significantly decreased in CD (D’Odorico et al., 2001; Kruidenier et al., 2003; Koutroubakis et al., 2004; Hengstermann et al., 2008). Therefore, the combination of excess ROS production and diminished antioxidant capacity might explain several pathophysiological aspects of CD (Alzoghaibi, 2013).

Plasma free thiols (R-SH, sulfhydryl groups) are generally considered as a robust measure of the overall *in vivo* reduction-oxidation (redox) status. Thiol groups are rapidly oxidized by reactive oxygen metabolites, thus systemic oxidative stress is associated with reduced plasma free thiol levels (Banne et al., 2003). Extracellularly, reduced free thiols are present in very low concentrations and the percentage of oxidized thiols is higher as compared to the intracellular environment (Turell et al., 2013). In plasma of healthy individuals, thiols predominantly occur as protein-embedded cysteine residues (Go and Jones, 2011). Circulating albumin harbors the largest pool of plasma free thiol groups, of which roughly 75% is present in the reduced state in healthy conditions (Hortin et al., 2008). Furthermore, low molecular weight (LMW) thiols, though scarcely present in the extracellular compartment, mainly circulate complexed with albumin (Turell et al., 2013). High concentrations of plasma free thiols, as potent antioxidants, are thus representative of a more favorable redox status *in vivo*.

Although plasma free thiols may pose a reliable reflection of systemic oxidative stress, concentrations of plasma free thiol groups have not yet been evaluated in IBD, nor in CD specifically. Similarly, to the best of our knowledge, the potential role of plasma free thiols as biomarker for intestinal health has not been addressed before. In contrast, the role of free thiols has been examined in a variety of other (inflammatory) disease conditions, including cardiovascular disease (CVD), type 2 diabetes, and renal failure (Banne et al., 2003; Kundi et al., 2015; Frenay et al., 2016; Koning et al., 2016; Cortese-Krott et al., 2017). In other studies, decreased plasma free thiols were associated with cardiovascular risk factors, including aging, elevated body-mass index (BMI), and alcohol consumption (Go and Jones, 2011; Khalili et al., 2015). Conversely, elevated concentrations of plasma free thiol groups were strongly associated with favorable CVD outcome (Frenay et al., 2016; Koning et al., 2016).

Learning more about the systemic redox status, as reflected by plasma free thiols, is imperative since plasma free thiols have potent antioxidant activity and might serve as a therapeutic target (Dröge, 2002). Furthermore, CD patients with latent disease who show signs of systemic oxidative stress might have increased susceptibility to disease exacerbations. Therefore, determination of plasma free thiol concentrations and analyzing their associations with clinical and biochemical disease parameters might provide valuable information regarding the extent of systemic oxidative stress in CD in remission. In the present study, we hypothesized that plasma concentrations of free thiol groups (R-SH) are decreased in CD patients as compared to healthy controls and negatively correlate with inflammatory biomarkers. In addition, we aimed to identify associations of CD-specific disease (activity) parameters with plasma free thiol concentrations.

## Materials and Methods

### Study Population

This study is an exploratory analysis from a study cohort that has previously been described elsewhere (Bourgonje et al., 2018). From March 2016 to April 2017, 51 patients with an established diagnosis of CD were included at the IBD outpatient clinic of the University Medical Center Groningen (UMCG), Groningen, Netherlands. All patients were ≥18 years of age and had an established diagnosis of CD existing for at least 1 year. Diagnosis was based on clinical, endoscopic and histopathological criteria (Lennard-Jones, 1989). At time of inclusion, most patients were in clinical remission and 39 of 51 (76.5%) patients were treated with conventional IBD therapy (i.e., thiopurines, mesalamine, TNF-antagonists or a combination of these drugs). The study was approved by the Institutional Review Board (IRB) of the UMCG (in Dutch: “Medisch Ethische Toetsingscommissie”) (IRB No. 14/291). All patients provided written informed consent in accordance with the Declaration of Helsinki (2013). Blood samples were obtained after patients provided written informed consent. In addition, plasma samples of 27 healthy, non-IBD controls were included for comparison, which were retrieved from a UMCG biobank containing pre-donation samples of living kidney donors [PSI-UMCG (IRB No. 08/279)]. Plasma samples from CD patients and non-IBD controls were obtained according to the same protocol.

### Data Collection

Demographic characteristics of all participants were registered, including age, sex, body-mass index (BMI, body weight divided by squared height), and smoking behavior. For the CD cohort, further disease-specific clinical characteristics were recorded, including the Montreal classification, maintenance medication (thiopurines, mesalamine, TNF-antagonists, or combination therapy), surgical history, and the Harvey-Bradshaw Index (HBI) as clinical disease activity index (Harvey and Bradshaw, 1980). Disease location according to the Montreal classification was recorded from the most recently performed endoscopic evaluation, which was completed at least within 12 months of serum analysis. CD patients with upper gastrointestinal disease or active perianal disease were not included in the study. Routine laboratory measurements were performed in all study participants, including hemoglobin (Hb), C-reactive protein (CRP), erythrocyte sedimentation rate (ESR), white blood cell count (WBC), platelet count, albumin, aspartate transaminase (AST), alanine transaminase (ALT), and creatinine (Roche Modular, Roche, Mannheim, Germany). The estimated glomerular filtration rate (eGFR) was calculated using the Chronic Kidney Disease Epidemiology Collaboration (CKD-EPI) equation (Levey et al., 2009). In CD patients, fecal calprotectin levels were measured by enzyme-linked immunosorbent assays (ELISA) (BÜHLMANN Laboratories AG, Switzerland) as routine measurement in the UMCG.

### Measurement of Plasma Free Thiols

Plasma samples were stored at -80°C until measurement of free thiols. Plasma free thiol groups were measured as previously described, with minor modifications (Ellman, 1959; Hu et al., 1993). Firstly, samples were 4-fold diluted with 0.1 M Tris buffer (pH 8.2). After transfer to a microplate, background absorption was measured at 412 nm with a reference measurement at 630 nm using a Varioskan plate reader (Thermo Fisher Scientific, Breda, Netherlands). After adding 20 μL 1.9 mM 5,5′-dithio-bis (2-nitrobenzoic acid) (DTNB, Ellman’s Reagent, CAS-number 69-78-3, Sigma-Aldrich Corporation, St. Louis, MO, United States) in phosphate buffer (0.1 M, pH 7.0), following an incubation time of 20 min at room temperature, free thiol groups were colorimetrically detected through a second sample absorbance measurement. Final free thiol concentrations were determined by parallel measurement of a L-cysteine (CAS-number 52-90-4, Fluka Biochemika, Buchs, Switzerland) calibration curve with a concentration range from 15.6 μM to 1,000 μM in 0.1 M Tris/10 mM EDTA (pH 8.2). Plasma free thiol concentrations were corrected for plasma albumin by dividing the concentrations, since albumin is the most abundant human plasma protein, and is the predominant source of thiols (Turell et al., 2013).

### Measurement of Inflammatory Biomarkers

Measurements of serum concentrations of inflammatory biomarkers of CD patients were performed as previously described (Bourgonje et al., 2018). In short, serum samples from all CD patients were collected and stored in 1 mL aliquots at -80°C. After thawing, samples were centrifuged for 3 min at 2,000 × *g* to remove remaining particulates. Measurement of serum levels of high-sensitive C-reactive protein (hsCRP), serum amyloid A (SAA), IL-17A, and VEGF was performed using a customized electrochemiluminescence (ECL) multiplex assay [Meso Scale Discovery (MSD)^®^, Meso Scale Diagnostics, Rockville, MD, United States]. ECL signals were fitted to a 4-parameter logistic model with 1/y^2^ weighting, ensuring a broad range of molecule detection. Serum concentrations of all detected molecules were determined by using calibration curves to which the ECL signals were back-fitted. Final concentrations were calculated using the MSD Discovery Workbench analysis software^®^. All concentrations were above the lower limit of detection (LLOD).

### Statistical Analysis

Baseline demographic and clinical characteristics were presented as means ± standard deviation (SD) or proportions *n* with corresponding percentages (%). Non-normally distributed data were presented as medians [interquartile range (IQR)]. Assessment of normality of continuous variables was performed using histograms, normal probability plots (Q-Q plots) and the D’Agostino and Pearson omnibus K^2^ normality test (D’Agostino and Pearson, 1973). Between-group comparisons for continuous variables were performed using independent sample *t*-tests or Mann–Whitney *U*-tests, while for categorical variables chi-square tests or Fisher’s exact tests were used, as appropriate. CD patients were subdivided into two groups of below- and above-average albumin-adjusted plasma free thiol concentrations and were compared for clinical and laboratory parameters. Subsequently, univariable and multivariable linear regression analyses were performed to identify parameters that were independently associated with albumin-adjusted plasma free thiols. Multivariable linear regression analysis was performed using backward selection (*P*_out_ > 0.05), including all significantly associated variables from the univariable analysis. Skewed variables were logarithmically transformed before entry into linear regression. Differences between disease locations according to the Montreal classification were statistically compared using the non-parametric Kruskal–Wallis test. Correlations between inflammatory biomarkers and albumin-adjusted plasma free thiols were established using the non-parametric Spearman’s correlation coefficient (ρ). Where appropriate, Bonferroni corrections were applied to correct for multiple testing. All data were analyzed using SPSS Statistics 23.0 software package (SPSS Inc., Chicago, IL, United States) and visualized using GraphPad Prism 5.0 (GraphPad Software, San Diego, CA, United States). Two-tailed *P*-values ≤ 0.05 were considered statistically significant.

## Results

### Study Population Characteristics

Demographic and clinical characteristics of CD patients (*n* = 51) and healthy controls (HC) (*n* = 27) are presented in Table 1. CD patients had a significantly lower mean age as compared to healthy controls (42.2 ± 12.2 vs. 51.2 ± 8.6 years), while no significant gender differences were observed (74.5% vs. 63.0% females). Furthermore, differences between CD and HC were observed for several laboratory parameters: CD patients had significantly lower levels of hemoglobin (*P* = 0.001), albumin (*P* = 0.003), and creatinine (*P* = 0.005), whereas ESR (*P* < 0.001), platelet counts (*P* = 0.02) and eGFR (*P* < 0.001) were significantly increased compared to HC.

**Table 1:** Baseline characteristics of Crohn’s disease patients (CD) and healthy controls (HC).

Variables	HC	CD	*P*-value
	*n* = 27	*n* = 51	
Plasma free thiols per gram of albumin (μmol/g)	17.9 ± 1.8	14.7 ± 2.4	**< 0.001**
Age (years)	51.2 ± 8.6	42.2 ± 12.2	**< 0.001**
Female, *n* (%)	17 (63.0)	38 (74.5)	0.29
BMI (kg/m^2^)	26.4 ± 3.3	25.5 ± 5.3	0.32
Current smoking, *n* (%)	7 (25.9)	6 (11.8)	0.11
**Laboratory measurements**			
Hemoglobin (g/dL)	14.5 ± 1.3	13.5 ± 1.3	**0.001**
CRP (mg/L)^∗^	1.1 [0.6; 1.5]	1.8 [0.6; 4.2]	0.16
ESR (mm/h)^∗^	3 [2; 4]	14 [7; 27]	**< 0.001**
WBC (× 10^9^/L)^∗^	6.6 [5.0; 8.3]	6.6 [5.4; 7.9]	0.73
Platelets (× 10^9^/L)^∗^	239 [198; 303]	290 [246; 321]	**0.02**
Albumin (g/L)	45.6 ± 2.7	43.7 ± 2.7	**0.003**
AST (U/L)	20 ± 4	22 ± 8	0.18
ALT (U/L)^∗^	20 [17; 21]	20 [12; 23]	0.89
eGFR (mL/min × 1.73 m^2^)	89 ± 12	100 ± 14	**< 0.001**
Creatinine (μmol/l)	78.4 ± 15.8	69.7 ± 10.5	**0.005**

*Data are presented as mean ± SD or proportions (*n*, %). ^∗^Skewed data are presented as median [interquartile range]. *P*-values were two-tailed and calculated using independent sample *t*-tests or Mann–Whitney *U*-tests, as appropriate. Significances are indicated in bold. CD, Crohn’s disease; HC, healthy controls; BMI, body mass index; CRP, C-reactive protein; ESR, erythrocyte sedimentation rate; WBC, white blood cell count; AST, aspartate transaminase; ALT, alanine transaminase; eGFR, estimated glomerular filtration rate.*

### Distributions of Albumin-Adjusted Plasma Free Thiols

**FIGURE 1:**
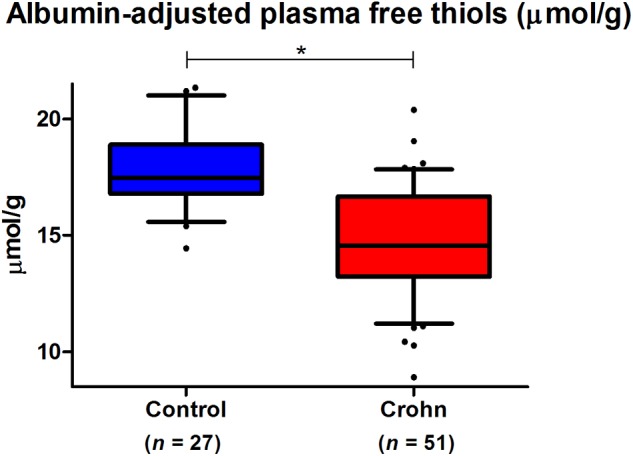
Albumin-adjusted plasma free thiols (μmol/g) are highly significantly reduced in Crohn’s disease (CD) patients compared to healthy controls (HC) (^∗^*P* < 0.001).

In both CD and HC, plasma free thiol (R-SH) concentrations were normally distributed (D’Agostino and Pearson omnibus K^2^ normality tests, *P* = 0.53 and *P* = 0.52, respectively). Albumin-adjusted plasma free thiols were significantly reduced in CD as compared to HC (with a mean of 14.7 ± 2.4 μmol/g for CD vs. 17.9 ± 1.8 μmol/g for HC, *P* < 0.001) (Figure 1). After adjustment for significantly different variables from Table 1, the difference in albumin-adjusted plasma free thiols remained statistically significant (*P* < 0.001).

Further baseline characteristics of the CD patient group are presented in Table 2, divided by the average concentration of albumin-adjusted plasma free thiols. CD patients with below-average albumin-adjusted plasma free thiols (<14.7 μmol/g) demonstrated significant differences for blood CRP levels and BMI, as compared to patients with above-average plasma free thiols per gram of albumin. Patients with below-average albumin-adjusted plasma free thiols had significantly increased CRP levels [3.6 (0.6; 7.0) vs. 1.4 (0.4; 2.6) mg/L, *P* < 0.05] and a significantly higher BMI (27.1 ± 5.2 vs. 23.6 ± 4.8 kg/m^2^, *P* < 0.05). No significant differences were observed for other documented variables. Clinical disease activity, as measured with the HBI, was not significantly different between patients with below- or above-average plasma free thiols. In the total CD cohort, median HBI score was 3 [1; 5], indicating that most patients had no active disease, based on these subjective disease activity scores.

**Table 2:** Baseline demographic, clinical and CD-specific characteristics compared between above- and below-average albumin-adjusted plasma free thiol concentrations (average: 14.7 ± 2.4 μmol/g).

Variables	Total CD cohort	Below-average thiols	Above-average thiols	*P*-value
	*n* = 51	*n* = 27	*n* = 24	
Plasma free thiols per gram of albumin (μmol/g)	14.7 ± 2.4	12.9 ± 1.6	16.8 ± 1.3	**< 0.001**
Age (years)	42.2 ± 12.2	42.2 ± 11.7	42.3 ± 13.0	0.98
Female, *n* (%)	38 (74.5)	20 (74.1)	18 (75.0)	0.94
BMI (kg/m^2^)	25.5 ± 5.3	27.1 ± 5.2	23.6 ± 4.8	**0.02**
Current smoking, *n* (%)	6 (11.8)	5 (18.5)	1 (4.2)	0.11
Prior surgery, *n* (%)	19 (37.3)	9 (33.3)	10 (41.7)	0.54
HBI score^∗^	3 [1; 5]	3 [1; 5]	3 [2; 5]	0.52
**Disease location, *n* (%)**				0.28
L1 (ileal)	17 (33.3)	9 (33.3)	8 (33.3)	
L2 (colonic)	11 (21.6)	8 (29.6)	3 (12.5)	
L3 (ileocolonic)	23 (45.1)	10 (37.0)	13 (54.2)	
**Medication, *n* (%)**				0.57
None	12 (23.5)	6 (22.2)	6 (25.0)	
Thiopurines	8 (15.7)	6 (22.2)	7 (29.2)	
Mesalamine	13 (25.5)	6 (22.2)	2 (8.3)	
TNF-antagonists	12 (23.5)	7 (25.9)	5 (20.8)	
Combination	6 (11.8)	2 (7.4)	4 (16.7)	
**Laboratory measurements**				
Hemoglobin (g/dL)	13.5 ± 1.3	13.5 ± 1.1	13.3 ± 1.5	0.71
CRP (mg/L)^∗^	1.8 [0.6; 4.2]	3.6 [0.6; 7.0]	1.4 [0.4; 2.6]	**0.04**
ESR (mm/h)^∗^	14 [7; 27]	17 [7; 27]	13 [6; 20]	0.36
WBC (× 10^9^/L)^∗^	6.6 [5.4; 7.9]	6.6 [5.2; 7.9]	6.9 [5.5; 7.9]	0.56
Platelets (× 10^9^/L)^∗^	290 [246; 321]	298 [252; 361]	275 [242; 311]	0.07
Albumin (g/L)	43.7 ± 2.7	43.7 ± 3.1	43.6 ± 2.2	0.92
AST (U/L)	22 ± 8	21 ± 5	24 ± 9	0.08
ALT (U/L)^∗^	16 [12; 23]	16 [14; 22]	16 [10; 27]	0.56
eGFR (mL/min × 1.73 m^2^)	100 ± 14	99 ± 15	101 ± 13	0.67
Creatinine (μmol/L)	69.7 ± 10.5	70.4 ± 8.8	68.8 ± 12.2	0.60
Fecal calprotectin (μg/g)^∗^	140 [42; 358]	74 [40; 325]	160 [74; 359]	0.08

*Data are presented as mean ± SD or proportions (*n*, %). ^∗^Skewed data are presented as median [interquartile range]. *P*-values were two-tailed and calculated using independent sample *t*-tests or Mann-Whitney *U*-tests, as appropriate. *P*-values < 0.05 were considered statistically significant. Significances are indicated in bold. CD, Crohn’s disease; BMI, body mass index; HBI, Harvey-Bradshaw Index; TNF, tumor necrosis factor; CRP, C-reactive protein; ESR, erythrocyte sedimentation rate; WBC, white blood cell count; AST, aspartate transaminase; ALT, alanine transaminase; eGFR, estimated glomerular filtration rate.*

### Associations of Albumin-Adjusted Plasma Free Thiols With Disease Parameters

Univariable linear regression analyses confirmed that only blood CRP levels and BMI were significantly associated with albumin-adjusted plasma free thiol concentrations. Multivariable linear regression analysis showed that only BMI was independently associated with albumin-adjusted free thiols (Table 3).

**Table 3:** Univariable and multivariable linear regression analyses of albumin-adjusted plasma R-SH in CD with clinical and biochemical parameters.

Plasma R-SH/gram of albumin	Univariable analysis		Multivariable analysis	
Variables	B coefficient^#^	*P*-value	B coefficient^#^	*P*-value
Age	–0.091	0.52		
Female	–0.044	0.76		
Current smoker	–0.099	0.49		
BMI	–0.345	**0.01^†^**	–0.337	**0.02^†^**
Prior surgery	0.072	0.62		
HBI^∗^	–0.087	0.58		
**Treatment**				
Thiopurines	0.248	0.08		
Mesalamine	–0.194	0.17		
TNF-antagonists	0.060	0.68		
**Laboratory measurements**				
Hemoglobin	–0.065	0.65		
CRP^∗^	–0.300	**0.03^†^**		
ESR^∗^	–0.173	0.22		
WBC^∗^	0.061	0.67		
Platelets^∗^	–0.134	0.35		
Albumin	–0.003	0.99		
AST	0.105	0.46		
ALT^∗^	–0.146	0.31		
eGFR	0.056	0.70		
Creatinine	0.015	0.92		
Fecal calprotectin^∗^	0.241	0.10		

*^∗^Skewed data have been logarithmically transformed before entry into analyses. ^#^Standardized beta (β) coefficient. ^†^*P*-values < 0.05 were considered statistically significant. Significances are indicated in bold. R-SH, free thiols; BMI, body mass index; HBI, Harvey Bradshaw Index; TNF, tumor necrosis factor; CRP, C-reactive protein; ESR, erythrocyte sedimentation rate; WBC, white blood cell count; AST, aspartate transaminase; ALT, alanine transaminase; eGFR, estimated glomerular filtration rate.*

**FIGURE 2:**
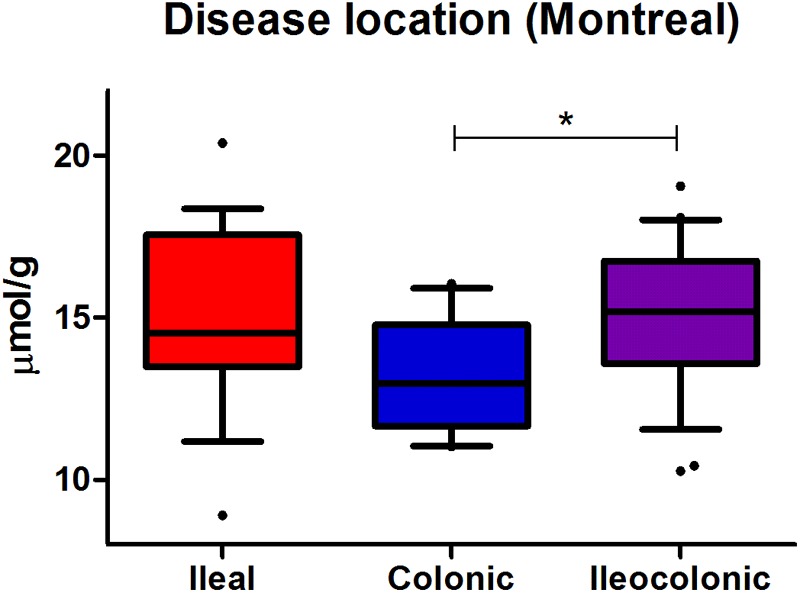
Albumin-adjusted plasma free thiols (μmol/g) are most prominently reduced in CD patients having solely colonic disease (colonic vs. ileocolonic CD, ^∗^*P* < 0.05), according to the Montreal classification of disease localization.

Furthermore, we observed an overall significant difference in albumin-adjusted plasma free thiols between different CD disease locations according to the Montreal classification (*P* < 0.05) (Figure 2). CD patients with solely colonic disease demonstrated significantly reduced plasma free thiol concentrations as compared to patients with ileocolonic disease (13.2 ± 1.8 μmol/g vs. 15.2 ± 2.2 μmol/g, *P* < 0.05). Patients with only ileal involvement (15.1 ± 2.8 μmol/g) showed concentrations comparable with patients having ileocolonic disease, but no significantly higher concentrations compared to patients with solely colonic CD.

**FIGURE 3:**
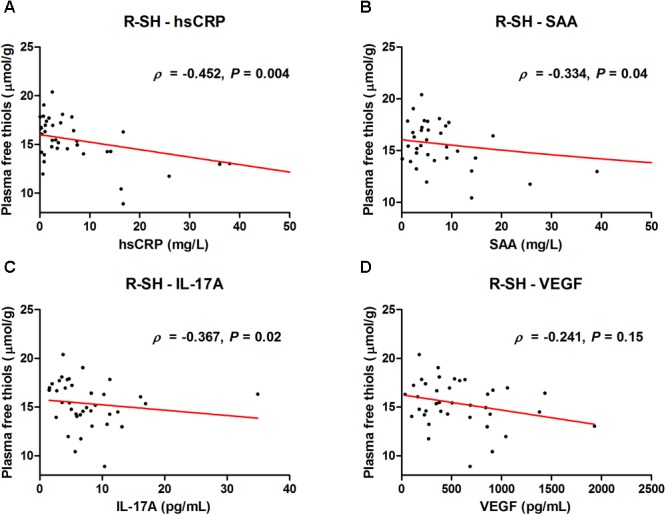
Serum levels of **(A)** high-sensitive CRP (hsCRP), **(B)** serum amyloid A (SAA), **(C)** interleukin-17A (IL-17A), and **(D)** vascular endothelial growth factor (VEGF) correlate with albumin-adjusted plasma free thiols (μmol/g).

Albumin-adjusted plasma free thiol concentrations significantly negatively correlated with several inflammatory biomarkers in CD (Figure 3). Among these, the strongest association was observed between albumin-adjusted plasma free thiols and high-sensitive C-reactive protein (hsCRP) (ρ = -0.452, *P* = 0.004) (Figure 3A). Additional significant correlations were demonstrated for serum amyloid A (SAA) (ρ = -0.334, *P* = 0.04) (Figure 3B) and interleukin-17A (IL-17A) levels (ρ = -0.367, *P* = 0.02) (Figure 3C). Serum levels of vascular endothelial growth factor (VEGF) also negatively correlated with albumin-adjusted plasma free thiols, though non-significantly (ρ = -0.241, *P* = 0.15) (Figure 3D).

## Discussion

In this study, we demonstrated that albumin-adjusted plasma free thiol concentrations are significantly decreased in CD in clinical remission as compared to healthy individuals. In the CD cohort, we found albumin-adjusted plasma free thiols to be inversely associated with CRP and BMI, i.e., patients with beneficial (above-average) concentrations of albumin-adjusted plasma free thiols had significantly lower CRP and lower BMI levels. The association with favorable disease status was further confirmed by the significantly negative correlations between plasma free thiol concentrations and the inflammatory biomarkers hsCRP, SAA, and IL-17A, and non-significantly with VEGF. Furthermore, we observed that CD patients with only colonic disease had significantly lower albumin-adjusted plasma free thiol concentrations as compared to patients with ileocolonic disease.

This is the first study to investigate the concentrations of plasma free thiols in a well-described cohort of CD patients in clinical remission. Our study was sufficiently powered to be able to detect a significant difference in albumin-adjusted plasma free thiols between CD and healthy individuals, even after adjustment for possible confounding factors. Furthermore, the observed reduction of plasma free thiol concentrations in these patients becomes an even more remarkable result when taking into account that most CD patients in this study had a normal HBI, indicating clinical remission.

In CD, an uncontrolled and persisting inflammatory response with disturbed intestinal homeostasis is pathologically represented by abundantly present inflammatory cells in the intestinal mucosa. Inflammation is intimately linked to oxidative stress, since the production and release of ROS by the various inflammatory cells is directly coupled to their immunological functions (Pereira et al., 2015). Furthermore, active intestinal inflammation in CD is characterized by increased vascular density and pathological tissue hypoxia, that may lead to increased ROS production through activation of targets of the hypoxia-inducible factor (HIF) transcription factor family (Biddlestone et al., 2015; Cummins and Crean, 2017). As such, the inflamed mucosa in CD is constantly exposed to the harmful effects of oxidative substances, eventually leading to extensive cell and tissue damage, as is characteristic for the disease. Oxidative stress is therefore considered to be one of the key effector mechanisms in CD pathogenesis and has been linked with many of its manifestations (D’Odorico et al., 2001; Taha et al., 2010; Alzoghaibi, 2013; Pereira et al., 2015). Additionally, ROS production by many different immune cells has been suggested to already occur before their infiltration into the intestinal mucosa, which is further substantiating their possible role in disease development (Beltrán et al., 2010). Moreover, this might partially explain the systemic concentrations of plasma free thiols as detected in the present study.

The plasma thiol signaling network has been subject to investigation in several other diseases, including CVD, acute and chronic renal failure, diabetes mellitus, familial hypercholesterolemia, and rheumatoid arthritis (Banne et al., 2003; Giustarini et al., 2005; Wlodek et al., 2006; Kundi et al., 2015; Qian et al., 2015; Ates et al., 2016; Koning et al., 2016; Cortese-Krott et al., 2017; Otal et al., 2018; Simsek et al., 2018). In all these studies, significant disturbances of the plasma thiol/disulfide balance were reported, with increased oxidized thiol forms (disulfides) and decreased free thiols, as compared to healthy subjects. Furthermore, the total plasma free thiol pool has been shown to be inversely related to age and cardiovascular risk factors (Jones et al., 2002; Go and Jones, 2011). This indicates that the differences we found may even be an underestimation, since our control group was slightly older. Additionally, in previous studies, serum free thiols have demonstrated to be significantly associated with favorable disease status and better prognosis, for example in CVD, liver failure, neurodegenerative diseases, and renal transplant recipients (RTRs) (Oettl et al., 2008; McBean et al., 2015; Frenay et al., 2016; Koning et al., 2016).

Extracellular antioxidant defense components available to counteract reactive species significantly differ from those found intracellularly, which mainly consist of the well-established antioxidant enzymes glutathione peroxidase (GSH), superoxide dismutase (SOD), and catalase (CAT). In human plasma, thiol-based plasma proteins predominate as major source of protection against oxidative stress, next to the common low molecular weight antioxidants including uric acid, ascorbic acid (vitamin C), and α-tocopherol (vitamin E) (Halliwell and Gutteridge, 1990; Motchnik et al., 1994; Turell et al., 2013). Albumin thiols are the most abundant thiols in human plasma (accounting for approximately 75% of the total thiol pool) and oxidized forms of albumin have been associated with many disease conditions (Anraku et al., 2013; Turell et al., 2013). Therefore, in the present study, we adjusted total free thiol concentrations to plasma albumin levels, which may more accurately represent systemic redox status.

This study is of particular importance since plasma thiols may be therapeutically targeted through the exogenous administration of thiol antioxidants (Deneke, 2000; Atkuri et al., 2007; Cortese-Krott et al., 2017). Various thiol antioxidants, such as *N*-acetylcysteine (NAC), GSH and other derivatives, have been proposed as treatment for restoring impaired thiol redox status in both animals and humans. For instance, NAC supplementation is known to lead to increased extracellular reducing capacity as directly available source of free sulfhydryl groups and to elevated levels of intracellular cysteine, providing a substrate for GSH synthesis (Atkuri et al., 2007). Another active thiol compound is L-2-oxothiazolidine-4-carboxylate (OTC), a lipophilic cysteine precursor that is converted intracellularly to free cysteine via the oxo-L-prolinase reaction (Meister, 1991; Porta et al., 1991). Although many similar compounds may also be effective, potential therapeutic interventions with thiol antioxidants should be developed with caution, since profound changes in thiol redox status could result in adverse effects. These might include derangement of transport signaling processes that are dependent on disulfide bonds in membrane proteins, single-electron oxidation of thiols forming thiyl radicals or a disequilibrium in the plasma thiol/disulfide balance that is typically comprised of more oxidized thiols under physiological circumstances (Kalyanaraman, 1995; Atkuri et al., 2007). Nevertheless, future thiol-based therapy, which has not been investigated yet, might be a promising strategy to beneficially modulate antioxidant bioavailability, as long as it is individually targeted to patients with pathological ROS production and, accordingly, reduced concentrations of plasma free thiols.

In the present study, we found CRP levels and BMI to be inversely associated with albumin-adjusted plasma free thiols. These results corroborate findings of a previous study in which free thiol concentrations were associated with clinical parameters in renal transplant recipients (Frenay et al., 2016). Similarly to our study, BMI appeared to be independently associated with plasma free thiol concentrations. Furthermore, this result is in agreement with another study that demonstrated protein-adjusted free thiols to be associated with a more favorable cardiovascular risk profile (Koning et al., 2016). In CD, obesity, as represented by an elevated BMI, has been associated with an increased risk of disease development, possibly through its association with (intestinal) inflammation (Khalili et al., 2015). Specifically for CD, it has been suggested that visceral adipose tissue (VAT), rather than total adiposity, is mainly affecting the risk of CD disease progression and largely contributes to the systemic inflammatory response (van der Sloot et al., 2017). In contrast to previous studies, the significance of the inverse relation between plasma free thiols and CRP levels was lost in multivariable analyses. Nonetheless, we were able to demonstrate significant inverse correlations between albumin-adjusted plasma free thiols and an array of inflammatory biomarkers, including the acute-phase reactants hsCRP, SAA, and the pro-inflammatory cytokine IL-17A, and a non-significant inverse correlation with VEGF. These data further confirm that inflammation and oxidative stress are two highly associated pathophysiological processes, represented by strong (negative) correlations of (anti)oxidant substances with higher concentrations of pro-inflammatory mediators in CD (Biasi et al., 2013).

Interestingly, albumin-adjusted plasma free thiol concentrations were most strikingly reduced in CD patients with only colonic disease. Indeed, previous literature shows that antioxidant capacity (as measured by enzymatic activity of SOD, catalase, and GSH) is relatively low in the colonic mucosa, already under physiological circumstances (Grisham et al., 1990). Furthermore, a considerable amount of evidence points toward increased mucosal ROS production in the inflamed colon that significantly correlates with CD disease activity (Kruidenier and Verspaget, 2002). Correspondingly, increased nitric oxide (NO) concentrations, belonging to the reactive nitrogen species (RNS), and being a major determinant of cellular redox status, have been found in colonic biopsy specimens of CD patients, including elevated expression of the NO-producing inducible nitric oxide synthase (iNOS) enzyme in the inflamed colonic mucosa (Boughton-Smith et al., 1993; Rachmilewitz et al., 1995; Dijkstra et al., 1998).

A strength of the present study is the inclusion of a healthy control group that enabled us to properly demonstrate differences in plasma free thiol concentrations with the CD study cohort. Furthermore, the study was sufficiently powered to detect significant differences between two extensively characterized cohorts of CD patients and healthy subjects. Despite this, some study limitations have to be taken into account. Most obviously, a greater sample size, including patients with more active disease, would have allowed us to more reliably establish associations between plasma free thiols and recorded disease-specific parameters. Furthermore, we were not able to examine plasma free thiols as potential biomarker for disease exacerbations, since endoscopic data close to the time of serum withdrawal were not available for this patient cohort. Similarly, no serial assessments were performed, which could have enabled us to examine plasma free thiols in relation to response to therapy or as risk factor for disease-related complications or hospitalization. In addition, the current study focused specifically on CD, but it would be interesting to study plasma free thiols in ulcerative colitis (UC) and compare putative correlations between both IBD entities.

In conclusion, we showed that albumin-adjusted plasma free thiols are significantly reduced in CD when compared to healthy subjects, especially in patients with colonic CD. Furthermore, we demonstrated above-average plasma free thiols to be associated with favorable outcome in CD, i.e., lower BMI and lower levels of inflammatory biomarkers (hsCRP, SAA, IL-17A, and VEGF). Future studies should focus on the clinical utility of plasma free thiols in CD as overall marker of systemic oxidative stress and aim to further unravel associations with established disease parameters. This study is of critical importance, since plasma free thiols form a potential substrate for therapeutic intervention. Hence, future antioxidant therapy might be a valuable strategy to ameliorate disease status in CD.

## Data Availability

The datasets generated for this study are available on request to the corresponding author.

## Ethics Statement

This study was carried out in accordance with the recommendations of Netherlands Code of Conduct for Research Integrity (2018), with which the University of Groningen complies. All subjects gave written informed consent in accordance with the Declaration of Helsinki (2013). The protocol was approved by the Institutional Review Board of the University Medical Center Groningen (full name in Dutch: “Medisch Ethische Toetsingscommissie”) (IRB No. 14/291).

## Author Contributions

AB, JvM, MB, KF, GD, and HvG were involved in conceptualization and study design. HvG and GD were responsible for funding acquisition and resources. JvM and GD obtained approval from the medical ethical board. AB, JvM, MvL, and MB collected all the study data. AB performed the data curation, data analysis, and visualization. AB wrote the first draft of the manuscript. All authors contributed to manuscript revision, read, and approved the submitted version.

## Conflict of Interest Statement

The authors declare that the research was conducted in the absence of any commercial or financial relationships that could be construed as a potential conflict of interest.
